# The role of preoperative opioid use in shoulder surgery—A systematic review

**DOI:** 10.1177/17585732211070193

**Published:** 2022-01-05

**Authors:** Omar A Al-Mohrej, Carlos Prada, Kim Madden, Harsha Shanthanna, Timothy Leroux, Moin Khan

**Affiliations:** 1 Division of Orthopaedic Surgery, Department of Surgery, 3710 McMaster University, Hamilton, Ontario, Canada; 2 Section of Orthopedic Surgery, Department of Surgery, 430300 King Abdullah Bin Abdulaziz University Hospital, Princess Nourah Bint Abdul Rahman University, Riyadh, Saudi Arabia; 3 Department of Health Research Methods, Evidence, and Impact, 3710 McMaster University, Hamilton, Ontario, Canada; 4 Division of Orthopedic Surgery, University of Toronto, Toronto, Ontario, Canada

**Keywords:** opioid, preoperative, shoulder surgery, total shoulder arthroplasty, arthroscopy

## Abstract

**Background:**

Emerging evidence suggests preoperative opioid use may increase the risk of negative outcomes following orthopedic procedures. This systematic review evaluated the impact of preoperative opioid use in patients undergoing shoulder surgery with respect to preoperative clinical outcomes, postoperative complications, and postoperative dependence on opioids.

**Methods:**

EMBASE, MEDLINE, CENTRAL, and CINAHL were searched from inception to April, 2021 for studies reporting preoperative opioid use and its effect on postoperative outcomes or opioid use. The search, data extraction and methodologic assessment were performed in duplicate for all included studies.

**Results:**

Twenty-one studies with a total of 257,301 patients were included in the final synthesis. Of which, 17 were level III evidence. Of those, 51.5% of the patients reported pre-operative opioid use. Fourteen studies (66.7%) reported a higher likelihood of opioid use at follow-up among those used opioids preoperatively compared to preoperative opioid-naïve patients. Eight studies (38.1%) showed lower functional measurements and range of motion in opioid group compared to the non-opioid group post-operatively.

**Conclusion:**

Preoperative opioid use in patients undergoing shoulder surgeries is associated with lower functional scores and post-operative range of motion. Most concerning is preoperative opioid use may predict increased post-operative opioid requirements and potential for misuse in patients.

**Level of evidence:**

Level IV, Systematic review.

## Introduction

The opioid crisis is a major public health issue that has resulted in widespread overdose and addiction with recent estimates suggesting that no less than 289 million opioid prescriptions are filled yearly in the United States.^1^ Recent studies suggest that at least 1.9 million Americans have developed opioid use disorder,^2,3^ and at least 530 people die weekly from opioid overdose.^4^ Over 50% of opioid-related deaths occur despite acceptable prescription of the medications with respect to medical board guidelines.^5^ Additionally, complications arising from opioid misuse and overuse result in significant healthcare expenses, estimated at an annual cost of $78.5 billion in US.^6^

Optimal pain control for patients with shoulder disorders or following surgical intervention is an area of ongoing research.^7^ Current practices of orthopedic surgeons are highly variable and opioids are commonly relied upon or prescribed without individualizing the needs of patients.^8^ As many patients expect to be pain-free postoperatively and satisfaction of treatment is associated with such an expectation, orthopedic surgeons have been keen to achieve this goal, resulting in overreliance on discharge opioid prescriptions. This is reflected in the fact that orthopedic surgeons file 7.7% of all US opioid prescriptions, representing the third-largest provider of opioids among clinicians.^9,10^

Opioid medications have traditionally been widely prescribed as a non-operative management of chronic joint pain. However, emerging evidence has shown that preoperative opioid use is associated with an increased risk of negative outcomes and increased postoperative demand for opioids following total joint arthroplasties.^11,12^ As such the utility of preoperative opioid use in such patients has come under significant scrutiny.^11,13,14^ Preoperative opioid use has been shown to be associated with increased readmission rates and risk of early revision among patients undergoing total knee or hip arthroplasty.^12^ Although increased revision rates and readmission were also evident in patients undergoing shoulder arthroplasty and rotator cuff repair,^15,16^ the effects of preoperative opioid use on revision surgery, readmission, and costs after shoulder surgeries are mixed. Some studies show no link between preoperative opioid use and complications, increased duration of stay, or readmission rates following shoulder arthroplasty.^17,18^

The objective of this systematic review is to evaluate currently available evidence on the association between preoperative opioid use in patients undergoing shoulder surgery with respect to perioperative clinical outcomes, postoperative complications, and postoperative dependence on opioids.

## Materials and methods

The study was conducted according to the Cochrane Handbook for Systematic Reviews^19^ and reported as per the Preferred Reporting Items for Systematic Reviews and Meta-Analyses (PRISMA) guidelines.^20^

### Search strategy

EMBASE, MEDLINE, PubMed, Cochrane Central Register of Controlled Trials (CENTRAL), and Cumulative Index to Nursing and Allied Health Literature (CINAHL) database were searched from date of inception to April 15th, 2021.

The search strategy, adapted to each database, included terms representing preoperative opioid use and its association with postoperative outcomes or opioid use. MeSH and EMTREE terms were used, along with free text, in several combinations to increase search sensitivity. We consulted with experts in the field, manually reviewed the reference lists of articles that fulfilled the eligibility criteria and used the “related articles” feature in PubMed. The search strategy was adapted in PubMed to search for articles published online ahead of print (Appendix 1).

### Eligibility criteria

In order to maximize potentially eligible data, no restrictions was made on publication date or follow-up. The criteria for inclusion were: (1) Studies on shoulder arthroplasty or scope procedures evaluating preoperative use of opioids; (2) Adult patients (3) Studies that reported at least one outcome related to shoulder arthroplasty or scope procedures; (4) Studies focused on postoperative opioid use; (5) any randomized or non-randomized studies were included.

The exclusion criteria were: (1) Studies that did not report on pre-operative opioid use; (2) Studies that included less than five patients; (3) Reviews and conference abstracts were excluded; (4) Studies without adequately reported functional outcomes or postoperative opioid use; (5) We excluded patients with functional pain syndromes. (6) Non-English language studies were excluded.

### Study screening

**Figure 1.**
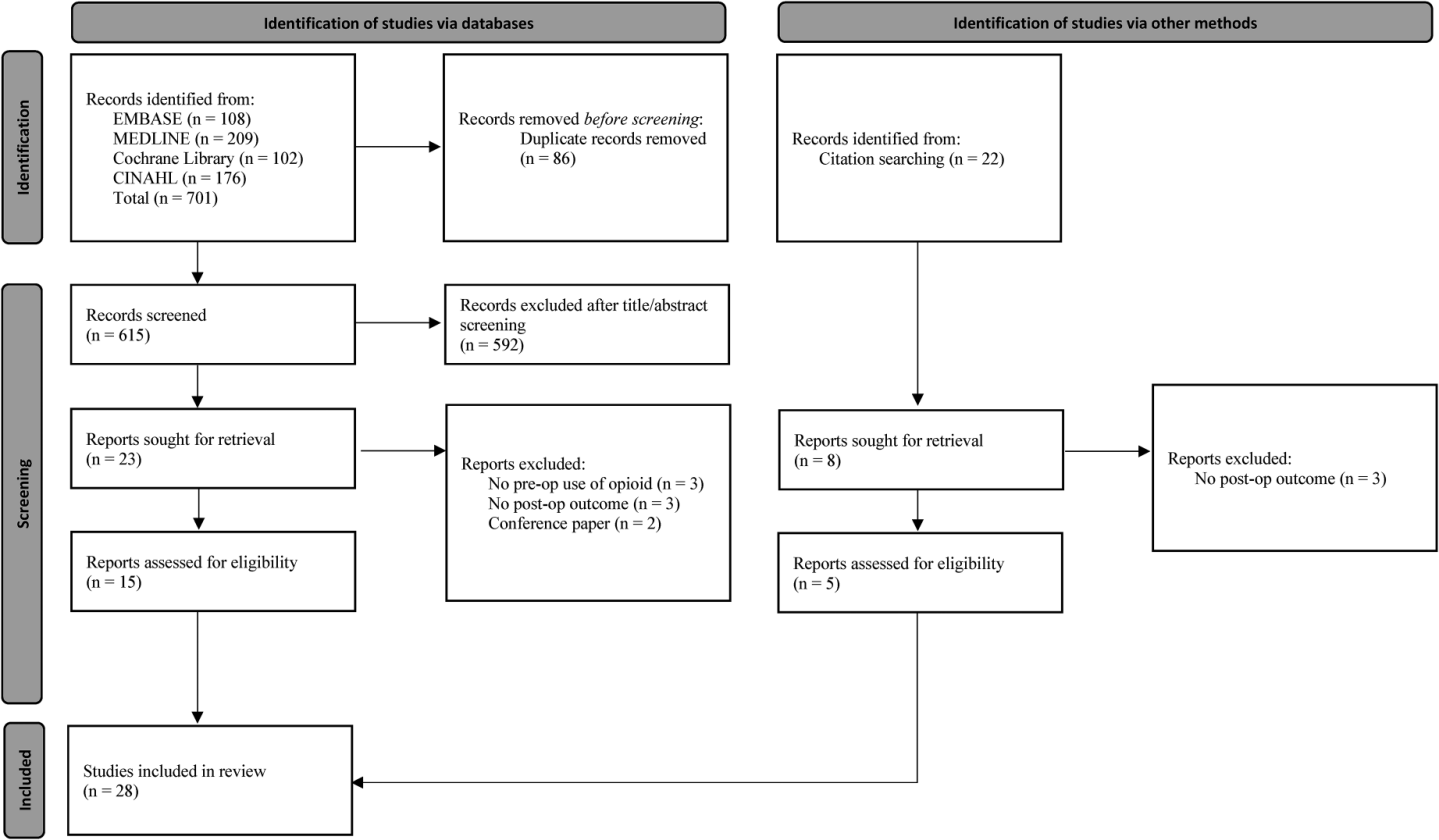
PRISMA flow diagram.

Study selection was performed by two authors (OAM, CP) using Covidence (Covidence systematic review software, Veritas Health Innovation, Melbourne, Australia). The selection of studies was performed in a stepwise manner, first by title and abstract, then full-text review. A resolve-by-consensus strategy was utilized for all discrepancies. If consensus could not be reached, a third senior researcher (MK) was consulted. Manual screening of the references of included studies was done to identify additional articles which may have eluded the initial search strategy (Figure 1).

### Extraction of data

Data were extracted by two reviewers (OAM, CP) independently from each other into a collaborative pre-defined data abstraction web-based spreadsheet (Google Sheets, 2021. California, United States: Google LLC.). To ensure accuracy, the results were audited by both reviewers (OAM, CP) and reviewed by the principal investigator (MK).

Extracted data included information on basic study characteristics (authors, publication year, country, journal of publication, study type), demographic data (age, sex, sample size, diagnosis, intervention type), preoperative opioid use, period of follow-up, study outcomes, and complications. The authors of included studies were contacted if there were any uncertainties regarding the data.

### Risk of bias and quality assessment

The Methodological Index for Non-Randomized Studies (MINORS) was used by two independent reviewers (OAM, CP) to assess risk of bias in the included studies.^21^ The MINORS scale assigns a score of 0, 1, or 2 for a list of 8 and 12 questions in non-comparative, and comparative studies, respectively.

### Statistical analysis

Descriptive statistics were calculated to reflect the frequency and percentage of abstracted study data. Using Cohen's Kappa (κ), inter-reviewer agreement at each stage of the screening process was calculated. Agreement was categorized a priori, as per Landis and Koch,^22^ with k of 0.81 to 1.0 for near perfect agreement; k of 0.61 to 0.80 for substantial agreement; k of 0.41 to 0.60 for moderate agreement; and k of 0.21 to 0.40 as fair agreement. Interobserver agreement for methodologic quality assessment was calculated using the intraclass correlation coefficient (ICC); a value of ≥0.65 was considered adequate.^23^

## Results

### Study characteristics

The search strategy retrieved 701 studies, of which 28 were identified to undergo full-text review. Twenty-one studies which met all of the inclusion criteria and were included in the final qualitative synthesis (Figure 1).^15–18,24–40^ There was substantial agreement for both title and abstract screening (Cohen's kappa = 0.71) and full-text review (Cohen's kappa = 0.72).

There was a near perfect level of agreement among quality assessment scores using the MINORS criteria (ICC, 0.93). The mean MINORS score for the included studies was 12 ± 0.7 out of 16 for non-comparative studies and 19.9 ± 1.3 out of 24 for comparative studies. Characteristics of included studies can be found in Table 1.

**Table 1.** Characteristics of included studies.

Study	Year	Study design	Level of evidence	Country	MINORS score	Age, mean ± SD	Sample size	Sex (female%)	BMI	Comorbid Conditions	Diagnosis	Operative Procedure	Follow-up preiod
Morris et al.	2015	Retrospective cohort study	III	US	19	68.5 ± 9.5	68	39 (57.3)	28.3 ± 7.35	No differences were found between the preoperative opioid group and the nonopioid group in smoking status, BMI, history of chronic back pain, depression, diabetes, or heart disease.	Rotator cuff arthropathy	rTSA	3.14 ± 1.7
Morris et al.	2016	Retrospective cohort study	III	US	18	66.5 ± 9.5	224	78 (34.8)	30.5 ± 6.0	No statistical differences were noted between the preoperative opioid group and the nonopioid group regarding diabetes, and heart disease, Patients with preoperative opioid use had a significantly higher prevalence of chronic back pain (52% vs. 30%; *P* = 0.003) and depression (13% vs. 4%; *P* = 0.04) and higher BMI (32 ± 7 kg/m2 vs. 29 ± 5 kg/m2; *P* = 0.01).	Glenohumeral joint osteoarthritis	aTSA	3.3 ± 1.2
Cuff et al.	2016	Prognostic case series	IV	US	12	63.3 years (range, 22 to 77 years)	181	79 (44)	NR	NR	Partial- or full-thickness rotator cuff tear	Arthroscopic rotator cuff surgery along with subacromial decompression	NR
Cheah et al.	2017	Retrospective cohort study	III	US	19	67.3	262	139 (53)	Opioid group had a significantly greater number of patients with a BMI greater than or equal to 30 compared with the non–opioid user cohort (*P* = 0.016).	All other characteristics were similar, with a noted trend that the opioid cohort had a slightly higher Charlson Comorbidity Index (*P* = 0.057)	NR	170 rTSA and 92 aTSA	2 years
Berglund et al.	2018	Retrospective cohort study	III	US	11	71 years (range, 30 to 90 years)	490	NR	NR	NR	OA without RCT, OA with RCT, Fracture sequelae, Avascular necrosis, Failed arthroplasty, Locked dislocation and Inflammatory arthritis	HA, aTSA, rTSA	47 months; range, 24 to 124 months
Grace et al.	2018	Retrospective analysis of prospective cohort study	II	US	21	median age was 68 years (range, 29–89 years)	119	56 (47.1)	30.1 ± 5.4	There was no significant difference in prevalence of depression (8.5% vs 5.6%, *P* = 0.53).	Osteoarthritis, Avascular necrosis, Proximal humeral fracture, Rotator cuff arthropathy	aTSA (37.0%), rTSA (63.0%).	NR
Menendez et al.	2018	Retrospective analysis of prospective cohort study	II	US	21	69 ± 8	415	253 (61)	30.8 ± 6.3	Patients reporting severe postoperative pain were more likely to have more self-reported allergies (3.4 vs. 1.5, *P* < 0.001), diabetes (22% vs. 11%, *P* = 0.005), comorbid diagnosis of major depression (37% vs. 21%, *P* = 0.001), and an ASA score ≥ III (30% vs. 20%, *P* = 0.031). The most prevalent comorbidities were hypertension (60%), hypercholesterolemia (39%), and depression (25%).	osteoarthritis (69), rotator cuff arthropathy (22)	aTSA (29%), rTSA (71%)	NR
Rao et al.	2018	Retrospective cohort study	III	US	12	84.1% (*n* = 3570) were aged ≥60 years	3996	2201 (51.9)	57.0% (*n* = 2176) had a BMI < 30	Hypertension was the most prevalent medical comorbidity (*n* = 2574 [69.0%]), and depression was the most common opioid use-related comorbidity (*n* = 604 [17.2%])	NR	Hemiarthroplasty/humeral head resurfacing 891 (21.0%), rTSA 923 (21.8%), aTSA 2429 (57.2%)	minimum of 1 year
Thompson et al.	2019	Retrospective cohort study	III	US	18	61.0 ± 11.8	73	36 (49.3)	32.2 ± 6.2	No significant differences were noted between the groups with respect comorbidities including DM, smoking, chronic pain syndromes, or mood disorders (including depression and anxiety).	primary glenohumeral osteoarthritis	aTSA	32.8 ± 10.3 months
Brock et al.	2019	Retrospective cohort study	III	US	21	69.0 ± 8.6	22,524	10,170 (45.5)	NR	Depression increased the risk for chronic postoperative opioid use, as did intravenous drug use for RCR but not TSA. Smoking and chronic lung disease were not significant predictors.	diagnosis codes used for TSA and RCR	TSA and rotator cuff repair	NR
Curtis et al.	2019	Retrospective cohort study	III	US	22	67.55 ± 11.15	138	73 (52.9)	NR	The opioid cohort included five patients with a medical history of myocardial infarction (versus 1 control subject; *P* = 0.014) and three with peptic ulcer disease (versus 0 control subject; *P* = 0.057). Differences between cohorts in all other comorbidities, were not statistically significant.	Osteoarthritis, Rotator cuff arthropathy, Chronic dislocation, fractures, AVN, Osteomyelitis, Periprosthetic infection, and Arthroplasty failure.	TSAs (69.1% reverse and 30.9% anatomic).	393 ± 299 days
Mayer et al.	2019	Prognostic case-control study	III	US	21	61.9	152	67 (44.0)	33.7	The chronic narcotic-use group had significantly more smokers than the non-narcotic group (*P* = 0.002); there were no other significant differences in comorbidities between groups. These comorbidities include: Asthma, COPD, Heart disease, HTN, DM, Depression/anxiety, OSA, and Kidney disease.	Osteoarthritis	aTSA	NR
Williams et al.	2019	Retrospective cohort study	III	US	19	58.4 ± 10.1	200	96 (48.0)	30.6 ± 6.1	Notable statistically significant differences between groups included increased frequencies of back pain, depression, use of antidepressants or anxiolytics, or both, and degenerative joint disease in the preoperative opioid group.	Full-thickness or partial-thickness tears of the supraspinatus tendon	Arthroscopic Rotator Cuff Repair	47.2 (15.1)
Khazi et al.	2019	Retrospective cohort study	III	US	20	Age < 25 yr = 2045 (42.6)	4802	1384 (28.82)	BMI ≥ 30 kg/m2 = 346 (7.21)	5.5% (*n* = 266) had a diagnosis of depression or anxiety, 7.1% (*n* = 339) had a diagnosis of fibromyalgia, and 0.3% (*n* = 15) had a diagnosis of Ehlers-Danlos syndrome	Shoulder instability	4268 (88.9%) underwent arthroscopic stabilization; 298 (6.2%), open Bankart procedures; 114 (2.4%), Latarjet procedures; and 122 (2.5%), other open procedures	minimum of 1 year
Kolade et al.	2020	Retrospective cohort study	III	US	13	68.6 ± 10.1	622	368 (59.5)	BMI was reported within bivariate analysis of average MME by patient characteristics for TSA patients with no differences between groups	Psychiatric disorders 217 (35); ASA II to IV 610 (98.1); Prior shoulder surgery 382 (62).	NR	rTSA (56%); TSA (43%); Revision rTSA (1.28%); Revision TSA (0.16%).	
Best et al.	2020	Prospective cohort study	II	US	12	57 ± 5.6	5621	2375 (42)	NR	Charlson comorbidity index 0.33 ± 0.68	NR	Primary TSA	NR
Sabesan et al.	2020	Retrospective cohort study	III	US	18	68.1 ± 9.4	162	94 (58)	30.2 ± 6.3	ASA class II to IV, 11.7% smokers	NR	60 aTSA; 22 rTSA (group 1) and 80 revision TSA patients (all had a revision to Reversed) (group 2).	
Jildeh et al.	2020	Retrospective cohort study	III	US	19	26.3 ± 11.7	340	85 (25)	27.5 ± 5.4	NR	Biceps tenodesis 12 (3.5); SLAP tear 105 (30.9); ALPSA, GLAD, or HAGL 13 (3.8); Bankart lesion 242 (71.2); Hill-Sachs lesion 150 (44.1); Reverse Hill-Sachs lesion 9 (2.6); Instability events 21 (97.3).	Shoulder arthroscopy with capsulorrhaphy including Bankart repair, arthroscopic SLAP repair, and limited arthroscopic debridement.	
Peratikos et al.	2020	Retrospective cohort study	III	US	21	61 (57 to 64)	1387	550 (40)	NR	Charlson comorbidity index, median (IQR) 0 (0 to 1); Severe comorbidity 47 (3); History of mood or anxiety disorder 398 (29); History of substance use disorder 82 (6), History of personality or trauma and stressor disorder 38 (3); History of tobacco use 232 (17)	NR	TSA	NR
Lu et al.	2020	Retrospective cohort study	III	US	21	56.82 ± 11.36	1242	505 (40.6)	31.06 ± 7.38	Depression 137 (11.0), Diabetes 113 (9.1), Heart disease 51 (4.1), Hypertension 313 (25.2), Degenerative joint disease 274 (18.1), Alcohol abuse 44, Smoking history 105 (6.5).	NR	Rotator cuff repair 298 (24.0); SLAP repair 25 (2.0); Capsulorraphy 102 (8.2); Biceps tenodesis 37 (3.0); Subacromial decompression 430 (34.6); Extensive debridement 220 (17.7); Limited debridement 130 (10.5).	minimum of 1 year
Farley et al.	2020	Retrospective cohort study	III	US	20	While statistically different, patient age breakdown was overall clinically similar among groups without an obvious trend.	214,283	92,123 (43.0)	NR	Significant differences in every examined comorbidity at baseline although small, they were universally higher in the high opioid use groups (*P* < 0.001 for all comparisons). These include: Obesity, Chronic kidney disease, Alcohol use disorder, Tobacco use, Hypertension, Coronary artery disease, Congestive heart failure, Hyperlipidemia, Rheumatic disease, Diabetes, Depression.	NR	Arthroscopic Rotator Cuff Repair ± biceps tenodesis, subacromial decompression, distal clavicle resection, these were overall similar between groups.	NR

The included studies were conducted between 2015 and 2020, with 17 (81.9%) studies published within 3 years of the search. Three of the studies were of level II evidence (*n* = 6155),^17,30,34^ one was level IV evidence (*n* = 181) and^38^ 17 were level III evidence, (*n* = 250,965). All of the included studies were performed in the United States.

This review includes a total of 257,301 patients. The median sample size was 378 participants (interquartile range: 167 to 2692). Of those, 110,771 (43.1%) were female (range: 39 to 92,123). The median age of the included participants was 66.9 (range: 22 to 90). Shoulder arthroplasty was evaluated in 14 studies, while shoulder arthroscopy was assessed in 6 studies. Brock et al. reported on outcomes of both types of procedures.^27^ The follow-up was reported in 10 studies, with a mean of 2.3 years. Regarding body mass index (BMI), the median was 30.5 kg/m^2^ (range: 27.5 to 32.2).

### Diagnosis

Partial- or full-thickness rotator cuff tears (RCTs), glenohumeral joint osteoarthritis with and without RCT, fracture sequelae, avascular necrosis (AVN), locked dislocation, inflammatory arthritis, osteomyelitis, and periprosthetic infection were reported pathology in the included studies. Seven studies did not report diagnosis of included patient populations.

### Opioid use

Preoperative: Of the included studies, 51.5% (*n* = 132,524) of the patients reported pre-operative opioid use. Also, 8 studies reported the opioid types, and 10 studies reported doses expressed as Oral Morphine Equivalent (OME) (Table 2).

**Table 2.** Preoperative opioid use.

Study	Year	Pre-op opioid use	Opioid type	Opioid dose (OME)	Post-op opioid use
Morris et al.	2015	32 (47.1)	NR	NR	NR
Morris et al.	2016	60 (26.8)	NR	NR	NR
Cuff et al.	2016	14 (8)	A prescription for 7.5 mg of hydrocodone and 325 mg of acetaminophen for postoperative pain management and was instructed to take 1 or 2 pills every 4 to 6 h as needed for pain during the first postoperative week		
Cheah et al.	2017	138 (52)	Short-acting opioids (oral hydrocodone, oxycodone, hydromorphone, or morphine) or long-acting opioids (extended release morphine, extended release oxycodone, methadone, or fentanyl patch)	Non–opioid users: 66.9 ± 41; Short-acting opioid users: 111.4 ± 127.3; Long-acting opioid users: 208.3 ± 135.3	NR
Berglund et al.	2018	174 (35.5)	NR	NR	Patients reporting preoperative narcotic use had a markedly higher incidence of opioid use at 1-year follow-up (29.1% versus 4.9%; OR, 8.320; 95% CI, 4.509 to 15.355; *P* < 0.001) and at final follow-up (35.1% versus 7.3%; OR, 6.877; 95% CI, 4.062 to 11.641; *P* < 0.001) compared with patients without preoperative use.
Grace et al.	2018	47 (39.4)	Acetaminophen and hydrocodone (55.3%), tramadol (21.3%), oxycodone immediate release (IR) (12.8%), acetaminophen and oxycodone (8.5%), oxycodone extended release (6.4%), acetaminophen and codeine (4.3%), methadone (4.3%), morphine extended release (4.3%), and hydromorphone (2.1%)	Opioid users were found to have significantly higher opioid requirements on the first postoperative day (60 OMEs vs 45 OMEs, *P* = 0.01) and the day prior to discharge (42 OMEs vs 15 OMEs, *P* < 0.001) than non-opioid users.	A significantly higher percentage of opioid users still required opioids 6 weeks after surgery, when compared with non-opioid users (71.0% vs 9.1%, *P* < 0.001)
Menendez et al.	2018	68 (16.4)	NR	NR	Patients with severe pain after surgery took more opioids (202 vs. 84 mg OMEs, *P* < 0.001; daily average: 73 vs. 42 mg OMEs, *P* < 0.001).
Rao et al.	2018	3182 (75%)	The most common opioid type prescribed was oxycodone (37%), followed by morphine (18%) and hydromorphone.	NR	Postoperatively, 92.6% used opioids in the early recovery period, and 38% to 42% used opioids in the later rehabilitation period
Thompson et al.	2019	26 (35.6)	NR	NR	NR
Brock et al.	2019	3992 (17.7)	NR	NR	1723 patients continued to have postoperative opioid use. Continuous chronic preoperative opioid use was the most significant risk factor for chronic postoperative use (odds ratio (OR) 4.84 to 39.75 for >6 M group, depending on the procedure).
Curtis et al.	2019	50 (36.2)	Tramadol only 7 (7.95), Tramadol and an additional opioid 4 (8), Narcotic 50 (36.23), Oxycodone 15 (30), Hydrocodone 33 (66), Morphine 3 (6), Hydromorphone 1 (2), Fentanyl 1 (2).	NR	Patients using opioids for pain management preoperatively were 4.7 times as likely to be on an opioid at 3 months postarthroplasty (confidence interval, 1.96 to 11.29; *P* = 0.001).
Mayer et al.	2019	27 (17.7)	NR	During the global postoperative period, chronic preoperative narcotic users had significantly higher cumulative OME compared to nonusers as inpatients and at 2 wk, 6 wk, and 12 wk (3209 Vs 1814; *P* = 0.003).	NR
Williams et al.	2019	44 (22.0)	NR	NR	On average (log-transformed), patients in the preoperative opioid group received 1.91 (95% confidence interval, 1.31–2.78) times more opioids over a postoperative course of treatment that was 2.73 (95% confidence interval, 1.62–4.59) times longer than patients who did not take opioids preoperatively
Khazi et al.	2019	1812 (37.7)	NR	NR	After the first postoperative month, patients in the preoperative opioid group had a significantly higher opioid prescription fill rate than patients in the N-OU group (*P* < 0.0001, Table 2)
Kolade et al.	2020	122 (20)	NR	47.4 (65.7)	Preoperative opioid use was correlated with higher inpatient opioid after TSA. Patients who received opioid prescriptions prior to surgery had 43% higher opioid consumption on average in the immediate postoperative period (*P* = 0.0013). The cohort of patients with preoperative opioid use had an average of 38.8 MMEs compared with 27.1 MMEs in the cohort of patients without a history of preoperative opioid use
Best et al.	2020	1571 (28)	Hydrocodone Short Acting 939 (60); Oxycodone Short Acting 386 (25); Codeine 54 (3.4); Oxycodone Long Acting 46 (2.9); Fentanyl Long Acting 31 (2.0); Hydromorphone Short Acting 27 (1.7); Morphine Long Acting 23 (1.5); Other 65 (4.1)	Oxycodone Short Acting 112 ± 261; Hydrocodone Short Acting 92 ± 116	Postoperative opioid use was found in 4424 (79)
Sabesan et al.	2020	In the primary arthroplasty cohort 19.5% patients were preoperatively dependent compared to 38.8% in the revision cohort.	NR	Mean preoperative TMEs of 115.2 (±245.6) for the revision arthroplasty group compared to the primary arthroplasty group (mean TMEs 31.5 ± 54.8). For the preoperatively naïve patients, the results were significantly lower, at 86.5 TMEs in the primary group and 115.1 for the revision group.	Postoperatively the revision group had significantly higher rates of postoperative dependence at 43.8% compared to 29.3% patients in the primary group. The OR for the type of surgery decreased after being adjusted for confounders, and its association with the outcome remained non-significant (Adjusted Odd Ratio (aOR) 1.27, 95%CI 0.59–1.74, *P* = 0.54). Preoperative dependence (aOR 6.44, 95%CI 2.89–14.4, *P* < 0.0001) was independently associated with postoperative opioid dependence ater adjustment.
Jildeh et al.	2020	Acute user 19 (5.6); Chronic user 32 (9.4); total 51 (15)	NR	NR	On average, chronic users filled 2.61 ± 3.53 prescription refills, acute opioid users filled 1.63 ± 2.09 prescription refills, and non-opioid users filled 0.57 ± 1.14 prescription refills.
Peratikos et al.	2020	318 (23)	NR	from 0 to 100	45 days post op 1243 (90); 18 months post op 322 (23)
Lu et al.	2020	184	Hydrocodone 214 (86.6), Oxycodone 9 (3.6), Hydromorphone 9 (3.6), Fentanyl (transdermal) 2 (0.8), Codeine 11 (4.45).	Perioperative daily OME: 20.5 ± 15.9, Perioperative OME above the threshold of 723: 31 (12.3)	Preoperative opioid use was the greatest predictor of postoperative opioid use (OR, 21.29, 95% CI, 12.2–37.15, *P* < 0.001).
Farley et al.	2020	120,569 (56.2)	Hydrocodone, oxycodone, oxymorphone, codeine, dihydrocodeine, morphine, hydromorphone, fentanyl, methadone, meperidine.	<1 OME (*n* = 16,468 [7.7%]); 1-<5 OMEs (*n* = 57,378 [26.8%]); 5-<10 OMEs; (*n* = 13,393 [6.3%]); >10 OMEs (*n* = 18,450 [8.6%])	NR

Postoperative: Fourteen studies (66.7%) reported a higher likelihood of opioid use at follow-up among those prescribed opioids preoperatively compared to preoperative opioid-naïve patients.

For instance, Grace et al.^17^ reported that opioid users were found to have significantly higher opioid requirements on the first postoperative day (60 OMEs vs 45 OMEs, *P* = 0.01) and the day prior to discharge (42 OMEs vs 15 OMEs, *P* < 0.001), than non-opioid users. Curtis et al.^15^ reported that patients using opioids for pain management preoperatively were 4.7 times as likely (95% CI 1.96 to 11.29; *P* = 0.001) to be on an opioid at 3 months post-arthroplasty. This was also found in a 1-year follow-up as per Berglund et al. in which patients reporting preoperative opioid use had a markedly higher incidence of opioid use (29.1% versus 4.9%; odds ratio, 8.320; *P* < 0.001).^40^

### Type of opioids

A wide variety of opioid medications were included, although they were reported in less than 50% of included studies. Cuff et al.^38^ reported only postoperative opioid regimen as a prescription for 7.5 mg of hydrocodone and 325 mg of acetaminophen during the first postoperative week. On the other hand, 4 studies reported preoperative use of opioids which included acetaminophen and hydrocodone, tramadol, oxycodone immediate release, acetaminophen and oxycodone, oxycodone extended release, acetaminophen and codeine, methadone, morphine extended release, and hydromorphone.^15,17,24,30^

### Comorbid conditions

In the majority of included studies reporting comorbidies, no statistical differences were noted between the preoperative opioid and non-opioid groups.^15–17,29,36,37^

However, Farley et al.^24^ identified significant differences in every examined comorbidity at baseline as they were universally higher in the high opioid use groups (*P* < 0.001 for all comparisons). Two studies linked between preoperative opioid use and prevalence of one of the following comorbidities: chronic back pain, depression, obesity, self-reported allergies and an ASA score ≥ III.^27,34^ Three studies did not report comorbid conditions.^28,38,40^

### Pain reporting, functional scores and range of motion (ROM)

Pain reporting and functional assessment scores were reported in eight studies (38.1%). The American Shoulder and Elbow Surgeons (ASES) score was the most commonly used (8 studies) followed by Visual Analogue Scale (VAS) for pain which was reported in 6 studies. Four studies assessed ROM at final follow up (Table 3).

**Table 3.** Pain reporting, functional scores and range of motion.

Study	Year	Functional assessment scores	Baseline ROM (active or passive)	ROM (active or passive) scores at final follow up	Functional scores (pre-op)	Functional scores (post-op)
Morris et al.	2015	Constant score, the American Shoulder and Elbow Surgeons (ASES) score, the Western Ontario Osteoarthritis Shoulder (WOOS) index, the Single Assessment Numeric Evaluation (SANE), and range of motion measurements.	Preoperative opioid group: Forward flexion 40 ± 37; Abduction 38 ± 36; External rotation 8 ± 16. Non opioid group: Forward flexion 43 ± 51; Abduction 42 ± 49; External rotation 9 ± 13.	Pre-oprative opioid group: Forward flexion 142 ± 30; Abduction 136 ± 39; External rotation 32 ± 16. Non opioid group: Forward flexion 147 ± 29; Abduction 145 ± 30; External rotation 27 ± 16.	Pre-oprative opioid group: total Constant score 14.9 ± 8.7; ASES 25.6 ± 13.1; WOOS 79.0 ± 14.0; SANE; 27.9 ± 26.4. Non opioid group: total Constant score 19.4 ± 13.3; ASES 37.4 ± 18.6; WOOS 67.4 ± 20.2; SANE; 27.9 ± 24.8.	Pre-oprative opioid group: total Constant score 58.8 ± 19.5; ASES 65.0 ± 26.4; WOOS 33.4 ± 31.6; SANE; 62.8 ± 32.9. Non opioid group: total Constant score 67.4 ± 18.5; ASES 75.8 ± 21.4; WOOS 22.3 ± 21.1; SANE; 62.1 ± 35.8
Morris et al.	2016	Constant score, the American Shoulder and Elbow Surgeons (ASES) score, the Western Ontario Osteoarthritis Shoulder (WOOS) index, the Single Assessment Numeric Evaluation (SANE), and range of motion measurements.	Preoperative opioid group: Forward flexion 73 ± 39; Abduction 70 ± 39; External rotation 11 ± 14. Non opioid group: Forward flexion 89 ± 37; Abduction 82 ± 36; External rotation 11 ± 15.	Pre-oprative opioid group: Forward flexion 155 ± 32; Abduction 153 ± 32; External rotation 45 ± 13. Non opioid group: Forward flexion 164 ± 13; Abduction 164 ± 14; External rotation 46 ± 13.	Pre-oprative opioid group: total Constant score 21 ± 16; ASES 33 ± 19; WOOS 76 ± 13; SANE 32 ± 28. Non opioid group: total Constant score 32 ± 17; ASES 44 ± 18; WOOS 62 ± 18; SANE 32 ± 24.	Pre-oprative opioid group: total Constant score 73 ± 20; ASES 83 ± 20; WOOS 19 ± 25; SANE 67 ± 35. Non opioid group: total Constant score 82 ± 12; ASES 89 ± 15; WOOS 11 ± 16; SANE 71 ± 36.
Cuff et al.	2016	VAS	NR	NR	NR	NR
Cheah et al.	2017	VAS, ASES	NR	NR	VAS pain scores between groups were similar, with a trend of higher scores in long-acting opioid users; ASES shoulder scores between groups were similar, with a trend of higher scores in opioid users.	VAS pain scores were higher in opioid users (4.0 ± 1.8 for nonusers, 4.9 ± 1.9 for short-acting users, and 6.0 ± 1.5 for long-acting users; *P* < 0.001); ASES scores: Analysis of variance showed similar preoperative and 2-year postoperative ASES results between both non–opioid users and opioid users in the TSA and rTSA groups.
Berglund et al.	2018	NR	NR	NR	NR	NR
Grace et al.	2018	VAS and ASES score	NR	NR	Opioid users reported worse pain (7 vs 4, *P* = 0.007) and ASES (32.8 vs 46.0, *P* = 0.003) scores in the preoperative period compared with non-opioid users.	Opioid users again reported more pain in the operative shoulder than non-opioid users 6 weeks after surgery (1 vs 0, *P* = 0.036), the magnitude of improvement from the preoperative visit to 6 weeks after surgery was similar between the 2 cohorts (4 vs 3, *P* = 0.16). Both the 6-week postoperative ASES scores (51.6 vs 56.2, *P* = 0.32) and the magnitude of ASES score improvement from preoperatively to 6 weeks postoperatively (18.8 vs 10.2, *P* = 0.11) were similar between the 2 groups.
Menendez et al.	2018	Preoperative ASES score	NR	NR	34.1 ± 16.5	NR
Rao et al.	2018	NR	NR	NR	NR	NR
Thompson et al.	2019	ASES scores, VAS scores, ROM, strength	Preoperative opioid group: FE ROM 90 (130; 20 to 150), strength 4 (2; 3 to 5); ER ROM 30 (90; −10 to 80), strength 5 (1; 4 to 5); IR ROM 30 (85; 0 to 85), strength 5 (1; 4 to 5). Non opioid group: FE ROM 110 (170; 0 to 170), strength 5 (3; 2 to 5); ER ROM 30 (80; 0 to 80), strength 5 (2; 3 to 5); IR ROM 45 (120; 0 to 120), strength 5 (1; 4 to 5).	Preoperative opioid group: FE ROM 135 (130; 50 to 180), strength 5 (2; 3 to 5); ER ROM 45 (60;0 to 60), strength 5 (1; 4 to 5); IR ROM 60 (150; 0 to 150), strength 5 (1; 4 to 5). Non opioid group: FE ROM 160 (185; 85 to 270), strength 5 (1; 4 to 5); ER ROM 45 (60; 25 to 85), strength 5 (0; 4 to 5); IR ROM 60 (60; 30 to 90), strength 5 (0; 4 to 5).	Preoperative opioid group: ASES 32 (61; 0 to 61); VAS 6 (8; 2 to 10). Non opioid group: ASES 42 (75; 5 to 80); VAS 5 (9; 1 to 10).	Preoperative opioid group: ASES 61 (96; 0 to 96); VAS 2 (10; 2 to 10). Non opioid group: ASES 91.7 (43.4; 56.6 to 100); VAS 0 (3; 0 to 3).
Brock et al.	2019	NR	NR	NR	NR	NR
Curtis et al.	2019	Numeric Rating Scale (NRS)	NR	NR	Scores were not significantly different between cohorts. NRS at rest: (F = 1.26; *P* = 0.26). NRS with activity: (*F* = 0.01; *P* = 0.93).	Postoperatively, the opioid cohort demonstrated mean resting pain score of 3.0 6 2.6, which was 1.6 points higher than that of the nonopioid cohort, 1.4 6 1.9 (*P* < 0.001).
Mayer et al.	2019	VAS	NR	NR	No other significant differences in preop VAS between both groups (7.0 vs 6.0 *P* = 0.10)	At 2 wk postoperatively, there was no statistically significant difference in VAS scores between chronic preoperative narcotic users and nonusers, although there was a trend toward higher VAS scores among narcotic users. At 6 and 12 wk, however, chronic narcotic users had significantly higher VAS scores.
Williams et al.	2019	ASES, SST, VAS and ROM	Preoperative opioid group: Forward flexion 120 ± 36; Abduction 110 ± 37; External rotation (side) 56 ± 18; External rotation (90) 74 ± 18. Non opioid group: Forward flexion 134 ± 31; Abduction 126 ± 34; External rotation (side) 63 ± 14; External rotation (90) 78 ± 15.	Preoperative opioid group: Forward flexion 147 ± 22; Abduction 148 ± 19; External rotation (side) 65 ± 15; External rotation (90) 83 ± 15. Non opioid group: Forward flexion 153 ± 13; Abduction 151 ± 15; External rotation (side) 67 ± 8; External rotation (90) 83 ± 11.	Pre-oprative opioid group: total Constant score 48.3 ± 15.6; ASES 32.1 ± 16.1; SST 4.1 ± 2.5; VAS 6.7 ± 2.2. Non opioid group: total Constant score 60.1 ± 17.4; ASES 47.6 ± 19.6; SST 6.1 ± 3.3; VAS 4.9 ± 2.6.	Pre-oprative opioid group: total Constant score 81.3 ± 14.1; ASES 68.4 ± 27.8; SST 7.5 ± 3.7; VAS 3.2 ± 3.0. Non opioid group: total Constant score 60.1 ± 17.4; ASES 47.6 ± 19.6; SST 6.1 ± 3.3; VAS 4.9 ± 2.6.
Khazi et al.	2019	NR	NR	NR	NR	NR
Kolade et al.	2020	NR	NR	NR	NR	NR
Best et al.	2020	NR	NR	NR	NR	NR
Sabesan et al.	2020	NR	NR	NR	NR	NR
Jildeh et al.	2020	NR	NR	NR	NR	NR
Peratikos et al.	2020	NR	NR	NR	NR	NR
Lu et al.	2020	American Shoulder and Elbow Surgeo), SANE, Constant score, SF-12 MCS, SF-12 PCS, pain VAS, VR-12 MCS, VR-12 PCS, and Veterans RAND 6D	NR	NR	American Shoulder and Elbow Surgeon form (36.6 ± 16.8 vs 46.5 ± 17.5), SANE (25.10 ± 19.05 vs 32.64 ± 19.01), Constant–Murley score (10.9 ± 5.34 vs 12.5 ± 4.99), SF-12 MCS (51.1 ± 12.0 vs 55.2 ± 8.74), SF-12 PCS (31.1 ± 5.21 vs 35.5 ± 7.03), pain VAS (6.23 ± 1.91 vs 5.06 ± 2.13), VR-12 MCS (52.6 ± 11.2 vs 57.7 ± 8.32), VR-12 PCS (33.5 ± 5.7 vs 38.1 ± 7.46), and Veterans RAND 6D (59 ± 8 vs 65 ± 8), for the OU and NOU cohorts, respectively	Both the OU and NOU groups showed statistically significant improvement—at the *P* < 0.05 level—in all PROMs after shoulder surgery; however, the OU group had significantly worse absolute outcome scores on all PROM measures compared with the NOU group (all *P* < 0.001)
Farley et al.	2020	NR	NR	NR	NR	NR

Grace et al.^17^ reported that opioid users had worse VAS (7 vs 4, *P* = 0.007) and ASES (32.8 vs 46.0, *P* = 0.003) in the preoperative period compared with non-opioid users. Morris et al.^37^ found that the non-opioid group had significantly greater preoperative forward flexion (average 16° greater; *P* = 0.005) and abduction (average 12° greater; *P* = 0.03). Although both groups significantly improved on ROM measurements from preoperative to most recent follow-up (*P* < 0.001), the opioid group demonstrated significantly lower ROM measurements, except for external rotation (*P* = 0.58), compared to the non-opioid group post-operatively.

### Complications and revision surgery

Complications reported among included studies were of heterogeneous conclusion. Best et al. found that preoperative opioid use is independently associated with significantly greater odds of 90-day readmission following primary TSA (OR 2.56, 95% CI: 1.99, 3.29, *p* < 0.001) and with an increase in hospital length of stay of 0.13 days (*p* = 0.001).^30^ On the other hand, Mayer et al.^18^ found that the differences in complication rates and readmission rates were not statistically significant, and no difference in length of stay between both groups was seen.

Again, Best et al.^30^ reported preoperative opioid use is independently associated with having revision surgery within 1-year (OR 2.35, 95% CI: 1.46, 3.79, *p* = 0.001), which is consistent with the report of Peratikos et al. who showed an 18-month surgical revision of 3% in the opioid group.^26^ (Table 4).

**Table 4.** Complications and revision surgery.

Study	Year	Complications	Revision surgery	Study conclusion
Morris et al.	2015	NR	NR	Although Preoperative range of motion measurements did not differ significantly between the 2 groups, Preoperative opioid use was associated with significantly lower preoperative scores for ASES score (*P* = 0.004) and WOOS index (*P* = 0.008). Non group had significantly better post-operative outcome scores, including Constant–Total (*P* = 0.035), ASES (*P* = 0.005), and WOOS (*P* = 0.013)
Morris et al.	2016	NR	NR	Patients with preoperative opioid use have a significantly lower preoperative baseline and achieve significantly lower final outcome scores after TSA compared with patients without a history of preoperative opioid use
Cuff et al.	2016	NR	NR	Preoperative narcotic use was also significantly predictive (*P* = 0.010) of high pain scores on postoperative day 1 and day 7 (*P* = 0.019)
Cheah et al.	2017	With respect to perioperative outcomes, length of stay, and 90-day complication and readmission rates were similar regardless of preoperative opioid use.	Reoperation (90 d) was non significant between the two groups	Preoperative opioid use in shoulder arthroplasty patients is common and associated with increased postoperative pain and opioid consumption while preoperative opioid use was not found to be associated with increased perioperative complications including mobilization, length of stay, discharge to a skilled nursing facility, readmission, or reoperation within 90 days of shoulder arthroplasty.
Berglund et al.	2018	NR	NR	Patients taking preoperative opioids had five times greater incidence of opioid use postoperatively and was nearly seven times more likely to be using opioids at an average of 4 years later. Narcotic usage did not markedly change from 1-year follow-up to final follow-up.
Grace et al.	2018	NR	NR	Daily and total opioid regimens prescribed after primary shoulder arthroplasty were similar between prior opioid users and nonusers despite large differences in their inpatient opioid requirements.
Menendez et al.	2018	NR	NR	The predictors of severe postoperative pain were greater number of self-reported allergies, preoperative chronic opioid use, lower American Shoulder and Elbow Surgeons score, and depression. Patients reporting severe pain took more opioids, stayed longer in the hospital, used postacute inpatient rehabilitation services more frequently, and were more likely to be high-cost patients.
Rao et al.	2018	NR	NR	Opioid usage in patients undergoing SA is widespread at 1 year, with three-fourths of patients having been dispensed at least one Rx
Thompson et al.	2019	Four complications occurred in the narcotic group (14%) and one (2%) in the control group (*P* = 0.05).	No surgical intervention was performed for any complication encountered.	Postoperatively, significant differences were noted between the narcotic and nonnarcotic groups regarding American Shoulder and Elbow Surgeons scores and visual analog scale scores, as well as forward elevation, external rotation, and all strength measurements (P, 0.01). The nonnarcotic group had markedly higher American Shoulder and Elbow Surgeons scores, better overall range of motion and strength, and markedly lower visual analog scale scores than the narcotic group.
Brock et al.	2019	NR	NR	By far the most important risk factor for chronic postoperative opioid use was chronic preoperative opioid use; however, this risk was significantly lower when chronic opioid users did not fill an opioid prescription in the three months before surgery.
Curtis et al.	2019	NR	NR	Patients who take opioids preoperatively are at risk for increased postoperative pain, continued opioid use at 3 months, and longer duration of opioid use after shoulder arthroplasty compared with non-opioid users.
Mayer et al.	2019	The differences in complication rates and readmission rates were not statistically significant, and there was no difference in length of stay between groups.	NR	Patients using chronic preoperative narcotic pain medication had significantly higher VAS scores and narcotic requirements after anatomic TSA.
Williams et al.	2019	NR	NR	Patients taking opioids preoperatively required a significantly greater quantity and longer duration of postoperative opioid therapy and did not ultimately reach the same level of functionality, as indicated by final follow-up outcomes scores
Khazi et al.	2019	NR	NR	Patients who were prescribed opioids between 1 to 3 months before surgery had the highest risk of prolonged opioid use following surgery
Kolade et al.	2020	Intraoperative complications 27 (4)	NR	Preoperative opioid use was correlated with higher inpatient opioid after TSA.
Best et al.	2020	Preoperative opioid use is independently associated with significantly greater odds of 90-day readmission following primary TSA (OR 2.56, 95% CI: 1.99, 3.29, *p* < 0.001). Preoperative opioid use was associated with an increase in hospital length of stay by 0.13 days (*p* = 0.001)	Preoperative opioid use is independently associated with having revision surgery within 1-year (OR 2.35, 95% CI: 1.46, 3.79, *p* = 0.001).	Preoperative opioid use is associated with increased rates of readmission, revision surgery and higher healthcare costs following primary total shoulder arthroplasty.
Sabesan et al.	2020	NR	–	For the preoperatively dependent cohorts that were assessed our subanalyses demonstrated these patients had over 6 times increase in postoperative dependence regardless of primary or revision surgery.
Jildeh et al.	2020	NR	NR	preoperative opioid use correlates with postoperative opioid demand
Peratikos et al.	2020	Nonhome discharge 45 (3); 30-d hospital readmission 25 (2); 90-d surgical site infection 29 (2).	18-mo surgical revision 46 (3).	Preoperative opioid users had longer length of stay, increased revision rates, higher spend, and persistent opioid use, which worsened with dose.
Lu et al.	2020	NR	Consumption of perioperative OME >723 was the strongest predictor of revision surgery (OR, 8.59, 95% CI, 2.12–34.78, *P* < 0.003) at 1 year	Patients with a history of preoperative opioid use can achieve significant improvements in patient-reported outcomes after arthroscopic shoulder surgery. However, preoperative opioid use negatively impacts patients’ level of satisfaction and is a significant predictor of pain and continued opioid usage
Farley et al.	2020	As average daily preoperative OMEs increased, so too did the odds of incurring a postoperative complication. Compared with opioid-naive patients, all opioid use groups had increased odds of postoperative emergency department visits and readmissions at 30 and 90 days.	Patients averaging 10 OMEs per day showed a 103% (odds ratio, 2.03 [95% CI, 1.62–2.54]; *P* < 0.001) increase in the odds of revision surgery compared with opioid-naive patients.	Preoperative opioid use was a risk factor for complications and revision surgery after arthroscopic RCR. We also observed a dose-dependent response between opioid use and postoperative complications

## Discussion

The association between preoperative opioid use and clinical outcomes is complex. The primary finding of this review is that the preoperative use of opioids is associated with inferior clinical outcomes and increased postoperative opioid use in patients undergoing shoulder surgeries.

It has been proposed that preoperative opioid use in orthopedic surgery increases the risk of chronic opioid use, thereby contributing to the ongoing “opioid crisis” in North America.^41^ Indeed, opioid use has become a central focus of orthopedic research in recent years.^42^ Preoperative opioid use has been identified as a predictor of prolonged postoperative use after many orthopedic procedures.^43,44^ Bartels et al.^45^ and Kim et al.^46^ identified multiple predictors of long-term opioid use during follow-up such as high in-hospital opioid use, procedure type, anesthesia type, age, and insurance type.

One of the problems that exists when analyzing the literature in this topic, is the lack of standardization of the definition of “chronic opioid use” leading to a wide variety in terminology used and outcome reporting suggesting the need of standardization.^47^ Nevertheless, we noted that there is a higher likelihood of ongoing opioid use at follow-up among those prescribed opioids preoperatively compared to preoperative opioid-naïve patients. Most of the studies included in this review report that those patients given opioids prior to their shoulder surgery were more likely to continue using opioids after surgery and reported higher pain scores and lower functional outcomes. Opioid stewardship is very important as it seems likely that preoperative opioid use leads to opioid tolerance and a diminished analgesic effect which ultimately leads to worse postoperative pain and greater opioid consumption.^48,49^

Although our study suggests that prescribing patients an opioid before surgery is associated with ongoing long-term opioid use, we cannot exclude the possibility that this is an artifact arising from underlying patient characteristics (e.g. patients with chronic pain conditions or psychiatric disorders).^50–53^ In a large retrospective study involving 29,827 arthroscopic rotator cuff repair patients, Westermann et al.^54^ found that those patients prescribed opioids preoperatively were more than 7 times more likely to continue using opioids after discharge. Other factors (e.g. psychiatric conditions and lower back pain) were also identified as contributing to ongoing opioid use. Although most of the studies showed no statistical differences were noted between the preoperative opioid and non-opioid groups regarding comorbidities, it is possible that the worst outcomes associated with preoperative use of opioids might be linked with certain comorbidities and/or the presence of chronic pain in this population, with the subsequent impact on clinical outcomes. However, there is an urgent need for standardized, evidence-based postoperative opioid prescribing protocols,^55^ as well as the development of strategies to reduce prolonged opioid use following surgery that consider a multimodal analgesic approach.^56^ This can be achieved by identifying patients preoperatively and try to use strategies to decrease their preoperative opioid use.^57^

Goplen et al.^43^ reported that total joint arthroplasty patients that received opioids preoperatively have worse overall pain and function than opioid-naïve patients. This was also found in patients undergoing ACL reconstruction, where the preoperative opioid group scored more poorly in patient-reported outcomes (at baseline and 1-year follow-up) and were more likely to fail to reach a patient acceptable symptomatic state.^58^ Chronic opioid use before cervical arthrodesis has also been associated with worse functional outcomes following surgery.^59^ In the context of shoulder surgery outcomes, most of the studies found that patients with preoperative opioid use had poor functional scores throughout the postoperative period. However, it is noteworthy that the preoperative opioid group tended to start with lower functional scores, so that the magnitudes of improvement did not differ greatly when comparing the preoperative opioid and non-preoperative opioid groups.^18,26,30^ In a subsequent work, Cozowicz et al. found that those patients with the most opioid prescriptions were at increased risk of treatment complications.^60^ Although this review was carefully designed to focus on functional outcomes, these data were missing from many of the included studies. Nevertheless, these results highlight important points regarding functional outcomes and ROM in patients undergoing shoulder surgeries, which develops earlier work questioning the benefit of prescribing opioids preoperatively to orthopedic surgery patients.

Recently, there has been a trend towards the use of non-opioid pain regimens favoring multimodal analgesia schemes. In fact, opioids are no longer a part of standard procedures in the treatment of athletes,^61,62^ and have been proposed as the second or third-line pharmacological option for the management of chronic painful joint conditions such as osteoarthritis.^63,64^ Multimodal analgesia can improve pain control and lessen reliance on opioids.^57,65^ Also, it was recently reported that a multimodal pain protocol could improve recovery (patient-reported) following arthroscopic shoulder surgery, with reduced opioid use relative to a conventional opioid-receiving group.^66^ Despite the importance that multimodal analgesia schemes might have in decreasing postoperative opioid use, future studies should ideally have as their key measure the improvements in patient-related outcomes instead of decreased opioid usage.^67^

### Limitations

There are several limitations in this review. First, we could not establish causality between preoperative opioid use and its effects on the outcomes of shoulder surgeries. This is due to many confounders, such as chronic pain, which were not adequately addressed in included studies. Furthermore, the indications of the preoperative opioid use were not mentioned since these medications might be taken for other causes of pain such as back pain or cancer pain.

Also, this review lacked any level I type of evidence. Because of this, some groups may have been imbalanced concerning gender, severity of back pain, mood disorders, and other chronic pain conditions.

Second, our analyses were constrained by the availability of published data as most of the studies included in this review did not include data on patient-reported outcomes, ROM, or strength. This was due to the older studies lacking access to the big data registries currently available, and the absence of certain information which was probably omitted during the surgeon's routine follow-up assessments. This limits the applicability of our results when shoulder surgeons have their preoperative counseling with their patients who are chronic users of opioids.

Despite these limitations, our findings are consistent with those reported in other orthopedics studies.^43,68–70^ Nevertheless, further research is required to better understand the influence that preoperative opioids have on postoperative outcomes in shoulder surgery.

Taken together, our findings agree with the previous orthopedic literature regarding preoperative use of opioids. This work and prior studies have identified major associations with preoperative opioid use: inferior outcomes, greater ongoing opioid use, depression, anxiety, and psychiatric conditions. Collectively, these data are likely to help guide clinical and surgical decision-making when planning orthopedic patient care protocols. Even if those patients given opioids preoperative are likely to experience an equal magnitude of improvement as those not given opioids. Also, postoperative programs and pain management counseling should be considered to help patients achieve adequate analgesia, thereby reducing the opioid burden.

## Conclusion

Our review suggests that in patients undergoing shoulder surgery, the preoperative use of opioids is associated with inferior clinical outcomes and increased postoperative opioid use and misuse. It is unclear to what degree this influence is due to the opioid use itself and to what extent it is related to comorbidities, severity of the preoperative condition leading to surgery, and/or chronic pain prevalence. Future prospective studies controlling for comorbidities, severity of the pathology and chronic pain, and OME are needed.

## Supplemental Material

sj-docx-1-sel-10.1177_17585732211070193 - Supplemental material for The role of preoperative opioid use in shoulder surgery—A systematic review Click here for additional data file. Supplemental material, sj-docx-1-sel-10.1177_17585732211070193 for The role of preoperative opioid use in shoulder surgery—A systematic review by Omar A Al-Mohrej, Carlos Prada, Kim Madden, Harsha Shanthanna, Timothy Leroux, and Moin Khan in Shoulder & Elbow
