# Supplementary material for: The role of preoperative opioid use in shoulder surgery—A systematic review
Source: Shoulder Elbow. 2022 Jan 5;15(3):250–73. doi: 10.1177/17585732211070193 (PMC10268141; doi:10.1177/17585732211070193)
Supplement: sj-docx-1-sel-10.1177_17585732211070193 - Supplemental material for The role of preoperative opioid use in shoulder surgery—A systematic review [file sj-docx-1-sel-10.1177_17585732211070193.docx]

**Search Terms**

**PICO:** pre-op opioid use and its impact on shoulder surgery (arthroplasty and arthroscopy) outcomes.

# **Medline: 209 hits**

1. Operative Time/ or operative.mp. or Surgical Procedures, Operative/
2. pre operative.mp.
3. pre-operat$.mp. [mp=title, abstract, original title, name of substance word, subject heading word, floating sub-heading word, keyword heading word, organism supplementary concept word, protocol supplementary concept word, rare disease supplementary concept word, unique identifier, synonyms]
4. 1 or 2 or 3
5. opioid.mp. or Analgesics, Opioid/
6. opiate.mp. or Opiate Alkaloids/
7. opio$.mp. or Opioid Peptides/
8. 5 or 6 or 7
9. Arthroplasty, Replacement, Knee/ or Arthroplasty, Replacement, Elbow/ or Arthroplasty, Replacement, Shoulder/ or arthroplasty.mp. or Arthroplasty, Replacement, Ankle/ or Arthroplasty, Subchondral/ or Arthroplasty, Replacement/ or Arthroplasty, Replacement, Finger/ or Arthroplasty/ or Arthroplasty, Replacement, Hip/
10. arthroscopy.mp. or Arthroscopy/
11. 9 or 10
12. 4 and 8 and 11
13. limit 12 to (English language and humans)

# **PUBMED: 106 hits**

1. pre operative
2. surger$
3. surgery$
4. ((surgery$) OR (surger$)) AND (pre operative)
5. opioi*
6. opioid analgesic
7. (opioid analgesic) OR (opioi*)
8. arthroplasty
9. arthroscopy
10. (arthroscopy) OR (arthroplasty)
11. ((((surgery$) OR (surger$)) AND (pre operative)) AND ((opioid analgesic) OR (opioi*))) AND ((arthroscopy) OR (arthroplasty))
12. ((((surgery$) OR (surger$)) AND (pre operative)) AND ((opioid analgesic) OR (opioi*))) AND ((arthroscopy) OR (arthroplasty)) Filters: Full text, English, Humans

# **EMBASE: 108 hits**

1. operative.mp.
2. pre-operativ*.mp. or surgery/
3. pre operati*.mp.
4. 1 or 2 or 3
5. opioid.mp. or opiate/
6. morphine/ or opiate/ or opioid analgesic.mp. or narcotic analgesic agent/
7. 5 or 6
8. revision arthroplasty/ or finger arthroplasty/ or knee arthroplasty/ or shoulder arthroplasty/ or abrasion arthroplasty/ or hip arthroplasty/ or total arthroplasty/ or Arthroplasty.mp. or total knee arthroplasty/ or replacement arthroplasty/ or arthroplasty/ or ankle arthroplasty/ or elbow arthroplasty/ or total shoulder arthroplasty/ or reverse shoulder arthroplasty/
9. shoulder arthroscopy/ or arthroscopy sheath/ or wrist arthroscopy/ or ankle arthroscopy/ or arthroscopy.mp. or hip arthroscopy/ or elbow arthroscopy/ or arthroscopy/ or knee arthroscopy/
10. 8 or 9
11. 4 and 7 and 10
12. limit 11 to (full text and human and English language)

# **COCHRANE LIBRARY VIA EMBASE: 102 hits**

1. operative.mp.
2. pre-operativ*.mp. or surgery/
3. pre operati*.mp.
4. 1 or 2 or 3
5. opioid.mp. or opiate/
6. morphine/ or opiate/ or opioid analgesic.mp. or narcotic analgesic agent/
7. 5 or 6
8. revision arthroplasty/ or finger arthroplasty/ or knee arthroplasty/ or shoulder arthroplasty/ or abrasion arthroplasty/ or hip arthroplasty/ or total arthroplasty/ or Arthroplasty.mp. or total knee arthroplasty/ or replacement arthroplasty/ or arthroplasty/ or ankle arthroplasty/ or elbow arthroplasty/ or total shoulder arthroplasty/ or reverse shoulder arthroplasty/
9. shoulder arthroscopy/ or arthroscopy sheath/ or wrist arthroscopy/ or ankle arthroscopy/ or arthroscopy.mp. or hip arthroscopy/ or elbow arthroscopy/ or arthroscopy/ or knee arthroscopy/
10. 8 or 9
11. 4 and 7 and 10
12. limit 11 to (full text and human and English language)
13. limit 4 to (full text and human and cochrane library and English language)
14. limit 7 to (full text and human and cochrane library and English language)
15. limit 10 to (full text and human and cochrane library and English language)

# **CINAHL=176 hits**

1. (MM "Arthroplasty") OR "arthroplasty" OR (MM "Hemiarthroplasty") OR (MM "Arthroplasty, Subchondral") OR (MM "Arthroplasty, Replacement") OR (MM "Arthroplasty, Reverse Total, Shoulder") OR (MM "Arthroplasty, Replacement, Shoulder") OR (MM "Arthroplasty, Replacement, Elbow") OR (MM "Arthroplasty, Knee, Unicompartmental") OR (MM "Arthroplasty, Replacement, Hip") OR (MM "Arthroplasty, Replacement, Knee") OR (MM "Arthroplasty, Replacement, Ankle")
2. Arthroscopy
3. (MM "Arthroscopy")
4. S1 OR S2 OR S3
5. (MH "Opioid Peptides") OR "opioid analgesics" OR (MH "Analgesics, Opioid") OR (MH "Narcotics")
6. (MM "Narcotic Antagonists") OR "opiates or opioids"
7. S5 OR S6
8. "pre-operative or pre-op or before surgery"
9. pre-operative
10. preoperative
11. S9 OR S10
12. S4 AND S7 AND S11
13. Peer Reviewed; English Language
